# Dose and Radioadaptive Response Analysis of Micronucleus Induction in Mouse Bone Marrow

**DOI:** 10.3390/ijms17091548

**Published:** 2016-09-13

**Authors:** Laura A. Bannister, Rebecca R. Mantha, Yvonne Devantier, Eugenia S. Petoukhov, Chantal L. A. Brideau, Mandy L. Serran, Dmitry Y. Klokov

**Affiliations:** Canadian Nuclear Laboratories, Radiobiology and Health, Chalk River, ON K0J1J0, Canada; rebecca.mantha@cnl.ca (R.R.M.); yvonne.devantier@cnl.ca (Y.D.); jenya.petoukhov@gmail.com (E.S.P.); bridech@ecolecatholique.ca (C.L.A.B.); mandy.serran@cnl.ca (M.L.S.); dmitry.klokov@cnl.ca (D.Y.K.)

**Keywords:** ionizing radiation, dose–response, mouse, C57BL/6, BALB/c, cytogenetic damage, radio-adaptive response, bone marrow, erythrocyte, micronucleus

## Abstract

Enhanced cellular DNA repair efficiency and suppression of genomic instability have been proposed as mechanisms underlying radio-adaptive responses following low-dose radiation exposures. We previously showed that low-dose γ irradiation does not generate radio-adaptation by lowering radiation-induced cytogenetic damage in mouse spleen. Since radiation may exert tissue-specific effects, we extended these results here by examining the effects of γ radiation on cytogenetic damage and proliferative index in bone marrow erythrocytes of C57BL/6 and BALB/c mice. In C57BL/6 mice, the induction of micronuclei in polychromatic erythrocytes (MN-PCE) was observed at radiation doses of 100 mGy and greater, and suppression of erythroblast maturation occurred at doses of >500 mGy. A linear dose–response relationship for MN-PCE frequencies in C57BL/6 mice was established for radiation doses between 100 mGy and 1 Gy, with departure from linearity at doses of >1 Gy. BALB/c mice exhibited increased MN-PCE frequencies above baseline following a 20 mGy radiation exposure but did not exhibit radio-sensitivity relative to C57BL/6 mice following 2 Gy exposure. Radio-adaptation of bone marrow erythrocytes was not observed in either strain of mice exposed to low-dose priming γ irradiation (single doses of 20 mGy or 100 mGy or multiple 20 mGy doses) administered at various times prior to acute 2 Gy irradiation, confirming the lack of radio-adaptive response for induction of cytogenetic damage or suppression or erythrocyte proliferation/maturation in bone marrow of these mouse strains.

## 1. Introduction

The classic Linear-No-Threshold (LNT) paradigm [1,2,3] asserts that the biological effects of ionizing radiation result from direct targeted damage to nuclear DNA. Stochastic risk estimates from low-dose, low-dose-rate exposures (i.e., <100 mSv) are derived by extrapolation from data obtained for high-dose radiation exposures. Cancer risk is viewed as being directly proportional to radiation dose and there is no recognized threshold dose below which cancer risk is not increased. However, LNT postulates are challenged by accumulating experimental evidence that non-DNA-targeted effects (NTEs), including radio-adaptive responses, bystander effects, genomic instability, low-dose hyper-sensitivity, abscopal effects and delayed reproductive death, play a crucial role in determining health effects following low-dose radiation exposure [4,5,6,7,8,9,10,11].

Radio-adaptive responses are a type of NTE manifested as a decrease in either acute detrimental radiation-induced or spontaneous biological health effects following exposures to low-dose radiation (mostly low-linear energy transfer (LET) radiation quality) [5,12,13,14]. Radio-adaptation has been reported for a variety of biological endpoints related to cellular toxicity and genotoxicity in vitro [15,16,17,18,19,20,21]. In relation to human radiation risk estimates, the most valuable experimental studies are those studying in vivo biological effects. Radio-adaptation or effects of low-dose irradiation have been demonstrated in vivo in mice for a variety of biological endpoints related to cellular toxicity and genotoxicity, oxidative stress, inflammation and immunity [18,22,23,24,25,26,27,28]. Increased lifespan, enhanced survival following high-dose radiation, decreased spontaneous and radiation-induced carcinogenic potential and suppression of tumour metastases have been identified as health risks in mice that are modified following chronic or acute low-dose irradiation [29,30,31,32,33,34,35]. Remarkably, earlier in vivo studies carried out in our laboratory showed that low-dose γ irradiation renders mice more resistant to radiation-induced or spontaneous age-related tumourigenesis expressed as increased tumour latency [29,30,36]. Enhanced cellular DNA double-strand break (DSB) repair and diminished genomic instability, potentially involving the *Trp53* tumour suppressor gene pathway, have been proposed as mechanisms that govern radio-adaptive responses and suppression of carcinogenesis following low-dose ionizing radiation exposures [26,28,30,36,37,38,39,40].

Although radio-adaptive responses are broadly acknowledged, they are by no means universal, and are influenced by a range of factors including radiation quality, dose and dose rate, cell type, tissue type and genetic makeup [14,41]. We previously demonstrated a lack of radio-adaptive response in mouse splenocytes for endpoints related to cytogenetic damage and DNA DSB repair. Mice exposed to low-dose γ radiation prior to acute high-dose γ radiation showed no reduction in the numbers of splenocyte DSBs, as measured by γH2AX foci (C57BL/6 mice) [42], or level of clastogenic damage, as measured by the Cytokinesis Block Micronucleus (CBMN) assay (C57BL/6 and BALB/c mice) [43].

We extended our previous results for mouse spleen tissue by analyzing potential radio-adaptive effects of low-dose γ radiation on micronuclei (MN) frequencies in bone marrow erythrocytes of C57BL/6 and BALB/c mice using the rodent micronucleus (MN) assay. This assay measures clastogenic and aneugenic damage in immature erythrocytes (polychromatic erythrocytes, or PCEs) and mature erythrocytes (normochromatic erythrocytes, or NCEs) of rodent bone marrow or peripheral blood [44,45,46,47]. In this study, mouse bone marrow MN-PCE frequencies were surveyed following radiation exposure. The ratio of polychromatic to normochromatic erythrocytes (the *P*/*N* ratio) was monitored in parallel as a measure of the erythrocyte proliferation or maturation index. Bone marrow samples in this study were derived from the same mice used to assess CBMN frequencies and cell survival endpoints in spleen tissue in our previous research [43], allowing direct comparison of the cytogenetic and cytotoxic effects of low-dose γ radiation and potential radio-adaptive responses in multiple tissues of radio-resistant and radio-sensitive mouse strains.

## 2. Results

### 2.1. Experimental Design and Rationale

**Figure 1:**
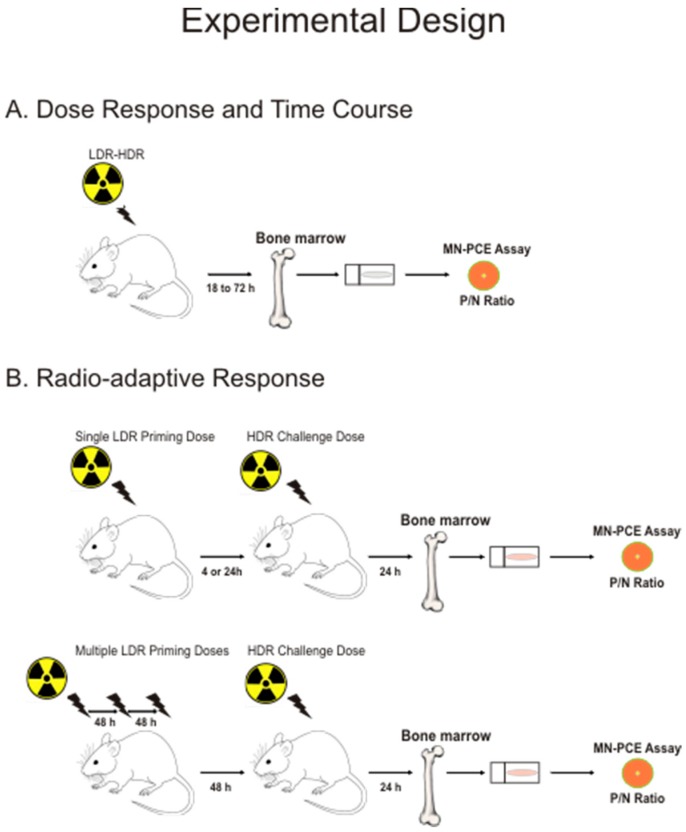
Overview of experimental design. Bone marrow cells were harvested from femora of C57BL/6 or BALB/c mice following total body in vivo LDR (low-dose radiation) or HDR (high-dose radiation) exposures to ^60^Co γ radiation. (**A**) For dose–response and time-course experiments, single acute doses of radiation (20 mGy to 2 Gy) were delivered to mice, and mice were euthanized 18 to 72 h post-irradiation; (**B**) For radio-adaptive response experiments, LDR was delivered as a single 20 mGy or 100 mGy priming dose (**B**, **upper panel**) or multiple 20 mGy priming doses (**B**, **lower panel**) at time intervals spanning four hours to six days prior to a subsequent HDR challenge dose, and mice were euthanized 24 h following the challenge irradiation. The induction of micronuclei in polychromatic erythrocytes (MN-PCE) frequency was measured in isolated bone marrow erythrocytes and the ratio of polychromatic: normochromatic erythrocytes (*P*/*N* ratio) was determined.

We initially characterized γ radiation dose–response and time-course relationships for MN-PCE frequencies and *P*/*N* ratios in mouse bone marrow to: (i) validate the sensitivity of the assay; (ii) examine dose-response relationships for radio-resistant and radio-sensitive mouse strains; and (iii) choose a proper time for endpoint measurements following a challenging high-dose exposure in the radio-adaptive response experiments. Thus, our study comprised two parts, one part examining dose–responses and kinetics for MN-PCE frequencies and *P*/*N* ratios, and a second part investigating radio-adaptive responses using conditions determined in the first part of the study. Low-dose γ radiation, given alone or as a priming dose prior to a subsequent acute challenging dose, was delivered to either mice or cells using similar dose regimes and time intervals to those previously used in our laboratory to demonstrate modulation of tumourigenesis in mice [29,37]. Female mice were used in this study to avoid sexual dimorphism in radiation response in keeping with our previous tumourigenesis studies that employed *Trp53* heterozygous and wild-type female mice. An overview of the experimental design is presented in Figure 1.

### 2.2. Dose Response for Bone Marrow Micronucleated Polychromatic Erythrocytes (MN-PCE) Frequency and Polychromatic/Normochromatic Erythrocyte (P/N) Ratio

**Figure 2:**
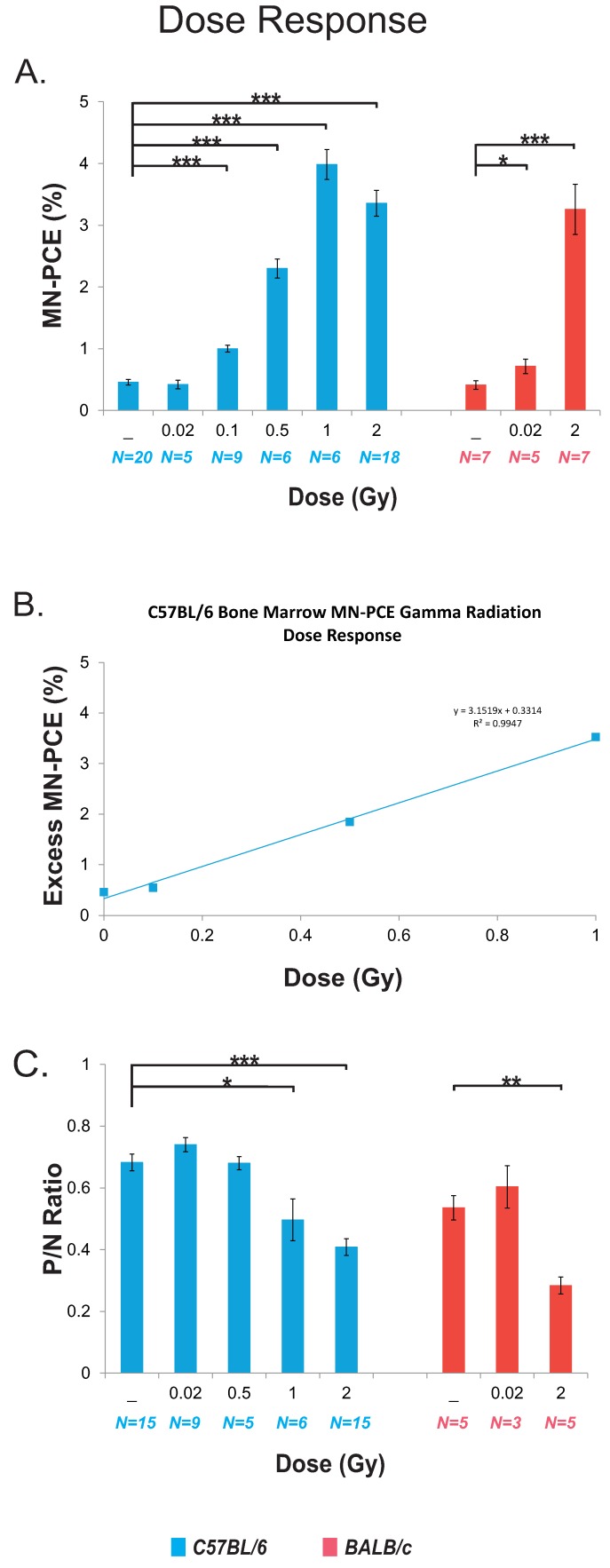
Dose–response for MN-PCE frequencies and polychromatic/normochromatic erythrocyte (*P*/*N*) ratios in mouse bone marrow. Radiation doses were delivered 24 h (1 and 2 Gy) or 28 h (20 mGy, 100 mGy and 500 mGy) prior to sacrifice. *N* = number of animals sampled per treatment group. Data points for C57BL/6 mice (blue columns) or BALB/c mice (red columns) represent the mean value of pooled samples; error bars indicate standard error. (**A**) Dose–response for MN-PCE induction; (**B**) Dose–response curve for excess MN-PCE (i.e., above spontaneous levels) between 100 mGy and 1 Gy for C57BL/6 bone marrow; and (**C**) Dose–response for *P*/*N* ratios. * *p* < 0.05, ** *p* < 0.005 and *** *p* < 0.0005 for unpaired Student’s *t*-test for irradiated cohorts in comparison to unirradiated control animals. 100 mGy, 500 mGy and 1 Gy radiation exposures were investigated for the C57BL/6 mouse strain only.

The dose–response relationship for C57BL/6 mice was investigated to determine the effects of a range of low to high acute doses of γ radiation on the induction of short-term (i.e., 24 to 28 h post-irradiation) clastogenic damage in PCEs. The mean endogenous MN-PCE frequency measured in unirradiated C57BL/6 control mice was 0.46% ± 0.045% (Figure 2A). MN-PCE frequencies did not differ significantly between unirradiated control and 20 mGy-irradiated animals (Figure 2A). MN-PCE frequencies were increased 2.2-, 5.0-, 8.7- and 7.3-fold following 100 mGy, 500 mGy, 1 Gy and 2 Gy irradiations, respectively, compared to unirradiated control animals (*p* < 0.0005 for all irradiated versus unirradiated control animals, Figure 2A). Maximum MN-PCE frequencies were observed following 1 and 2 Gy radiation exposures (3.98% ± 0.24% and 3.35% ± 0.21%, respectively) with no statistically significant difference between the MN-PCE frequencies at these two doses, indicative of a dose threshold between 500 mGy and 1 Gy for the induction of MN in PCEs. The shape of the dose–response curve for excess MN for C57BL/6 mice (i.e., above spontaneous levels) between 100 mGy and 1 Gy was best described by a linear fit; using regression and correlation analyses the dose–response equation was obtained as follows: F_excess MN-PCE (%)_ = 3.15*D* + 0.33, *R*^2^ = 0.99; where *D* = radiation dose in Gy (Figure 2B). Corresponding bone marrow *P*/*N* ratios of irradiated C57BL/6 mice were measured to monitor the dose–response relationship for suppression of erythrocyte turnover (progenitor proliferation and maturation). The mean endogenous *P*/*N* ratio measured in unirradiated control mice was 0.68 ± 0.027 (Figure 2C). No significant differences from unirradiated controls animals occurred at doses ≤500 mGy. Depression of the *P*/*N* ratio by 1.4-fold (*p* = 0.006) and 1.7-fold (*p* < 0.0005) ensued following 1 and 2 Gy irradiations, respectively, compared to unirradiated control animals (Figure 2C).

MN-PCE frequencies and *P*/*N* ratios were also examined for bone marrow of BALB/c mice exposed to acute γ radiation doses of 20 mGy and 2 Gy (28 and 24 h post-radiation, respectively). Baseline bone marrow MN-PCE frequencies (0.41% ± 0.071% for BALB/c mice; Figure 2A) and *P*/*N* ratios (0.54 ± 0.039 for BALB/C mice; Figure 2C) in unirradiated mice were not statistically different between the two mouse strains. In contrast to C57BL/6 mice, MN-PCE frequencies were increased 1.7-fold in BALB/c mice irradiated with a single 20 mGy radiation dose in comparison with unirradiated control animals (Figure 2A), and this increase was of marginal statistical significance (*p* = 0.042). The *P*/*N* ratio in BALB/c bone marrow following 20 mGy irradiation was not statistically different from the control ratio (Figure 2C). The MN-PCE frequency was increased 7.9-fold (*p* < 0.0005 in comparison to unirradiated mice) in BALB/c mice exposed to 2 Gy radiation, similar in magnitude to the induction of DNA damage observed for C57BL/6 mice following 2 Gy irradiation (Figure 2A). The *P*/*N* ratio of BALB/c mice was decreased 1.9-fold in comparison to the endogenous control ratio following irradiation with 2 Gy (Figure 2C).

### 2.3. Kinetics of Bone Marrow MN-PCE Induction and P/N Ratio Suppression Following 2 Gy Irradiation

**Figure 3:**
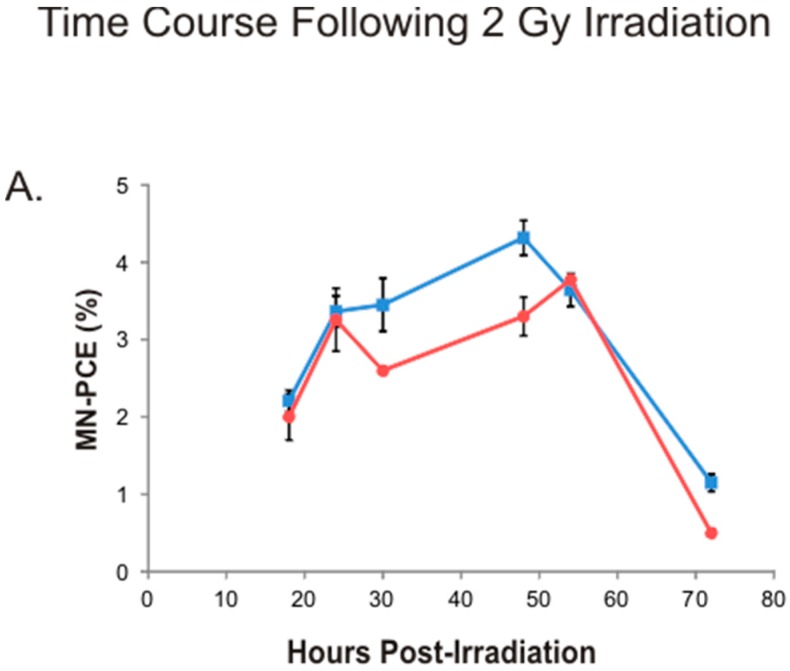

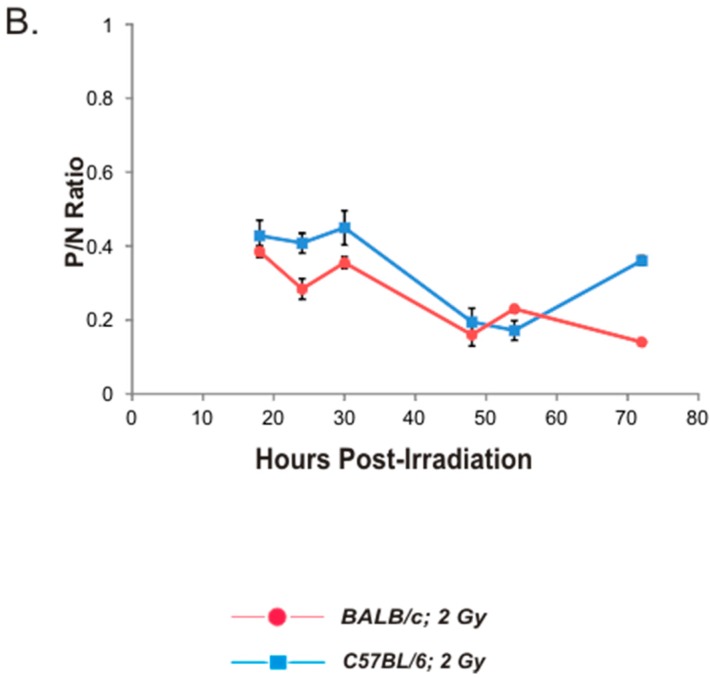
Time course analysis of MN-PCE frequencies and *P*/*N* ratios in mouse bone marrow following 2 Gy radiation exposure: Bone marrow MN-PCE frequencies (**A**) and *P*/*N* ratios (**B**) following 2 Gy irradiation of C57BL/6 (blue square symbols) or BALB/c (red circle symbols) mice. Data points represent the mean value of pooled samples; error bars indicate standard error. For C57BL/6 data analysis, six animals were sampled per treatment group, with the exception of the 24 h time point group comprised of 18 animals for MN-PCE analysis (reproduced from Figure 2) and 15 animals for *P*/*N* ratio determination (reproduced from Figure 2). For BALB/c data analysis, two animals were sampled per treatment group, with the exception of the 24 h time point group comprised of seven animals for MN-PCE analysis (reproduced from Figure 2) and five animals for *P*/*N* ratio determination (reproduced from Figure 2). Statistical analysis comparing C57BL/6 and BALB/c treatment cohort samples was not performed due to limited number of BALB/c samples for the majority of time points.

Time course experiments were conducted with C57BL/6 mice and a limited number of BALB/c mice to monitor the kinetics of formation and clearance of MN-PCE and modification of *P*/*N* ratios in bone marrow following acute high-dose γ irradiation. Mice were exposed to a 2 Gy dose and bone marrow MN-PCE and *P*/*N* ratio endpoints monitored 18 to 72 h post-irradiation. Data for MN-PCE frequencies and *P*/*N* ratios are shown in Figure 3. MN-PCE frequencies following the 2 Gy radiation dose were highest in C57BL/6 mice 48 h post-irradiation (9.4-fold above the baseline control frequency) and declined steeply between 54 and 72 h post-irradiation (Figure 3A). *P*/*N* ratios following 2 Gy irradiation of C57BL/6 mice reached a nadir between 48 and 54 h post-irradiation and began to recover by 72 h post-irradiation (Figure 3B) in the same time frame as PCEs with MN began to be cleared from bone marrow of irradiated mice (Figure 3A). The kinetic profile of MN increase in PCEs of BALB/c mice post-irradiation was similar to that of C57BL/6 mice, with the highest MN-PCE frequencies present 54 h post-irradiation (a 9.2-fold increase above the endogenous frequency) and a steep decline occurring between 54 and 72 h post-irradiation (Figure 3A). The maximal MN-PCE frequency observed in BALB/c mice at 54 h post-irradiation (3.8% ± 0.025%) was not statistically different from the maximal MN-PCE frequencies observed for C57BL/6 mice. Post-radiation temporal changes in *P*/*N* ratios in BALB/c mice were similar to those found for C57BL/6 mice (Figure 3B).

### 2.4. Radio-Adaptive Response for Bone Marrow MN-PCE Frequency and P/N Ratio

**Figure 4:**
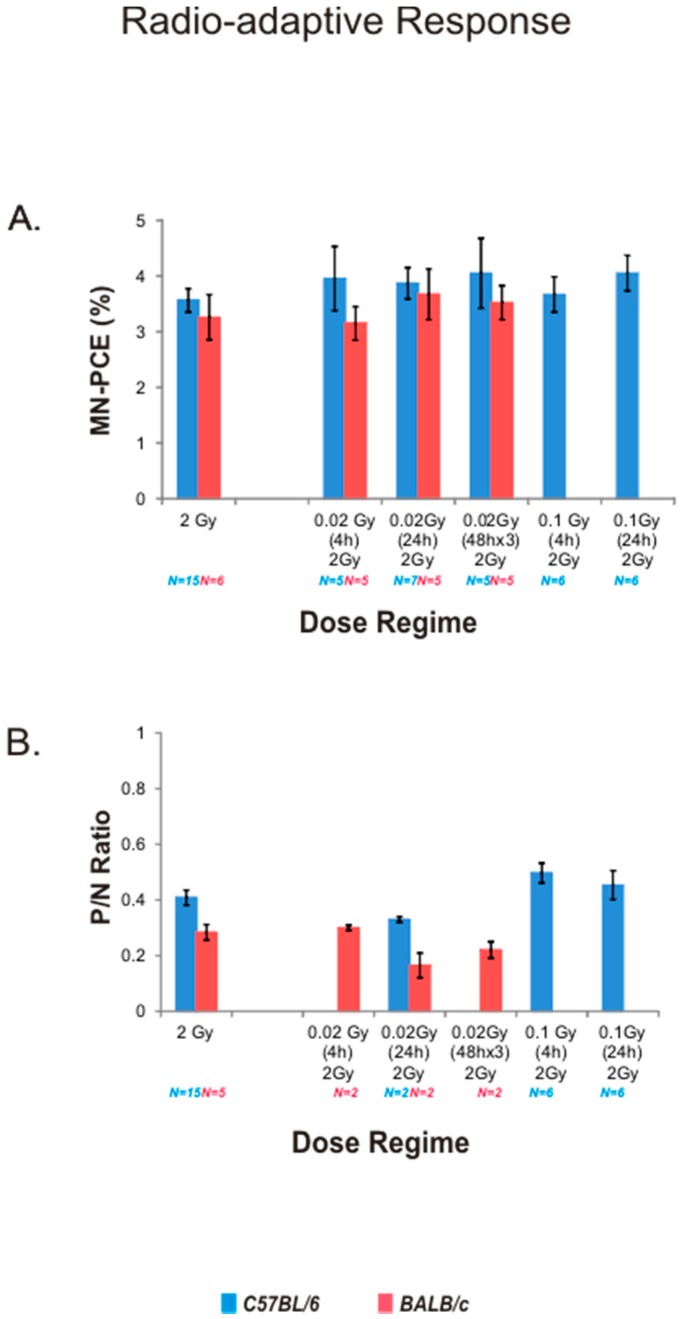
Radio-adaptive response analysis of MN-PCE frequencies and *P*/*N* Ratios in mouse bone marrow. Single or multiple LDR priming doses were delivered to C57BL/6 mice (blue columns) and BALB/c mice (red columns) prior to a 2 Gy challenge dose. Mice were euthanized 24 h post challenge dose and MN-PCE frequencies (**A**) and *P*/*N* ratios (**B**) were measured. Treatment groups receiving a 2 Gy radiation dose alone served as control groups (reproduced from Figure 2). *N* = number of animals sampled per treatment group. Data points represent the mean value of pooled samples; error bars indicate standard error.

Potential radio-adaptive responses occurring in vivo in bone marrow of C57BL/6 mice for modulation of MN-PCE frequencies and *P*/*N* ratios following acute high-dose γ radiation were assessed. In the first set of experiments conducted with C57BL/6 mice, a potential radio-adaptive priming dose (20 mGy irradiation with dose rate 0.5 mGy/min or 100 mGy with dose rate 10 mGy/min) was delivered as a single acute dose 4 or 24 h prior to the 2 Gy challenge dose, or as three separate 20 mGy doses (for a total dose of 60 mGy) six, four and two days prior to a 2 Gy challenge dose (Figure 1B). MN-PCE frequencies (Figure 4A) and *P*/*N* ratios (Figure 4B) were not altered significantly in C57BL/6 mice receiving any of the priming dose regimes in comparison to mice receiving the 2 Gy dose only. A single or multiple 20 mGy priming dose(s) was also given to BALB/c mice before a 2 Gy challenge dose to examine potential radio-adaptive responses in the bone marrow of this mouse strain. As observed for C57BL/6 mice, none of the priming dose regimes for the 20 mGy priming dose tested significantly affected MN-PCE frequencies (Figure 4A) or *P*/*N* ratios (Figure 4B) following acute challenge irradiation.

**Figure 5:**
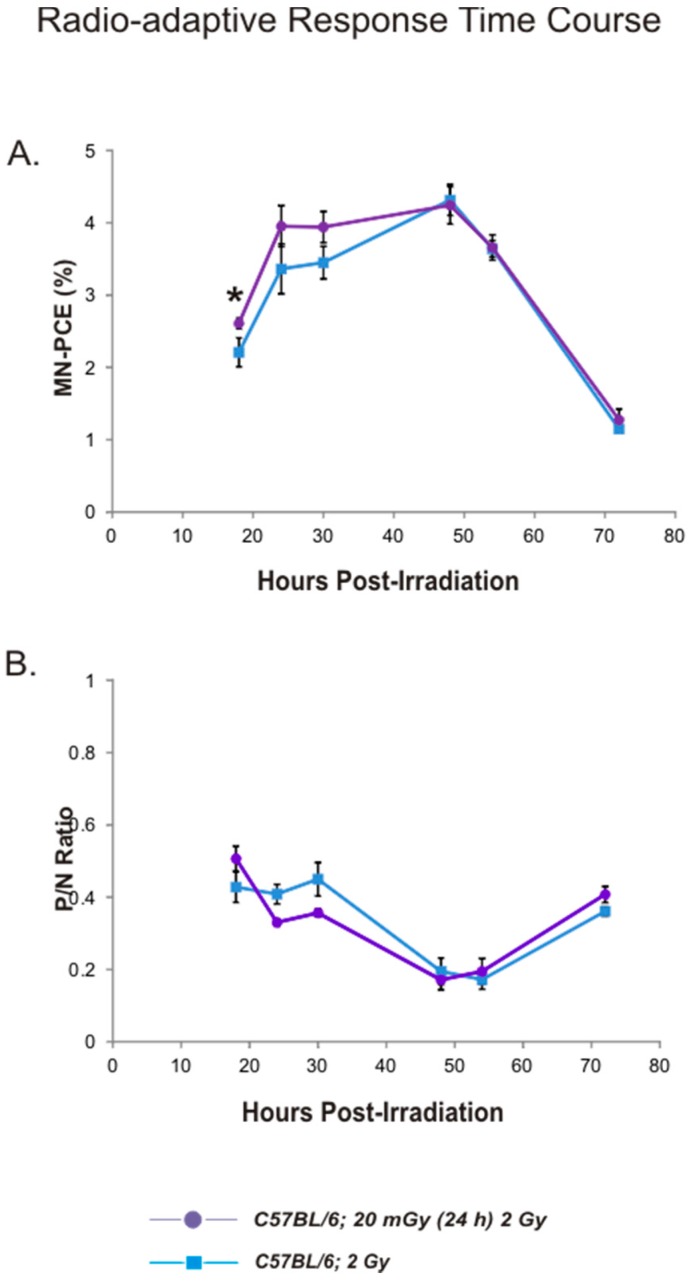
Radio-adaptive response time course analysis of MN-PCE frequencies and *P*/*N* ratios in mouse bone marrow. MN-PCE frequencies (**A**) and *P*/*N* ratios (**B**) following in vivo 20 mGy priming and 2 Gy challenge irradiations (purple circle symbols) or 2 Gy irradiation only blue (square symbols; data reproduced from Figure 3). Data points represent the mean value of pooled samples; error bars indicate standard error. Six animals were sampled per treatment group, with the exception of the 24 h time point, comprised of 18 animals for MN-PCE analysis (reproduced from Figure 2), 15 animals for *P*/*N* ratio determination (reproduced from Figure 2) and seven animals for the 2 Gy dose preceded by the 20 mGy priming dose treatment. * *p* < 0.05 for unpaired Student’s *t*-test.

It was feasible to assume that a potential radio-adaptive response to the priming exposures may have been masked by perturbations in the kinetics of 2 Gy-induced MN-PCE frequencies, if such perturbations had occurred. Therefore, in the second set of experiments, a time course spanning 18 to 72 h post-radiation was conducted with C57BL/6 mice to monitor MN-PCE frequencies and *P*/*N* ratios in mice that received a 20 mGy priming dose (delivered 24 h prior to the challenge dose) in comparison to mice that received the 2 Gy dose alone. In contrast to a radio-adaptive effect, a modest synergistic effect was observed in mice that received a 20 mGy priming dose, with a trend towards modestly increased MN frequencies (1.1- to 1.2-fold in the first 30 h post-radiation, Figure 5A). Statistical significance between the two experimental cohorts was observed only for the 18-h time point. No differences in MN-PCE and *P*/*N* ratios were witnessed between mice with and without the priming irradiation between 48 and 72 h following 2 Gy irradiation (Figure 5A,B). A trend for a steeper decline in *P*/*N* ratio for mice that received a 20 mGy priming dose in relation to those that received only 2 Gy radiation was observed for time points up to 48 h post-radiation (Figure 5B); however, no statistically significant differences were found for these time points.

## 3. Discussion

Radio-adaptive responses increase lifespan and suppress carcinogenesis in animal models, identifying this type of NTE as an important consequence of low-dose radiation exposure. However, radio-adaptive responses are highly variable, influenced not only be radiation quality, dose and dose rate, but also varying across cell types, tissues, systems, organisms and individuals [41,48,49]. Further investigation is warranted to define the biological conditions under which radio-adaptive responses do and do not prevail, and to determine underlying associated molecular processes. A multiple endpoint, multiple tissue approach for examining the biological effects of low-dose γ radiation exposure, including potential radio-adaptive responses, has been undertaken in our studies. We previously demonstrated a lack of radio-adaptive response for low-dose γ radiation in splenocytes of C57BL/6 and BALB/c mice for endpoints related to cytogenetic damage and DNA DSB repair [42,43] and confirmed higher frequencies of radiation-induced MN in splenocytes of BALB/c mice in comparison to C57BL/6 mice [43]. In this follow-up study, bone marrow from the same mice exposed to various irradiation regimes, including the ones allowing us to test for radio-adaptive responses, were analyzed to measure the dose–response and kinetic profile of MN-PCE induction and depression of erythroblast proliferation/maturation index and to examine cells for the manifestation of radio-adaptive responses in either of these endpoints.

The bone marrow MN-PCE assay measures DNA damage arising from clastogenic or aneugenic insult incurred in progenitor erythroblasts [50,51,52]. Increased bone marrow MN-PCE frequencies above baseline values are indicative of MN formed in immature newly formed erythrocytes, during the final cellular divisions, typically from damage incurred within 24 to 48 h from the sampling time [44,45,46,47]. The bone marrow *P*/*N* ratio reveals cellular turnover of PCEs and NCEs following short-term cellular damage and recovery post-treatment by replacement of erythrocytes in the marrow with undamaged progenitor cells.

In laboratory mice following radiation exposures at doses that do not delay erythroid cell cycle and maturation processes (i.e., doses ≤ 0.5 Gy low-LET radiation), an increase in the frequency of MN-PCE in bone marrow becomes apparent five to 16 h post-exposure, with the peak of maximum damage occurring 24 to 48 h following exposure [53,54,55,56,57]. The majority of MN present in cells following high-dose radiation exposure are extra-nuclear DNA fragments arising from acentric chromosome fragments originating from unrepaired or misrepaired DSBs [58]. Previous studies examining the dose–response relationship for γ and X-ray exposures and mouse bone marrow MN-PCE support a linear or linear-quadratic function for exposures to low to moderate doses of ionizing radiation (e.g., 0.1 to 0.5 Gy) [54,56,59,60,61,62,63]. More damaging radiations (>1 Gy) have been shown to prolong the erythroblast cell cycle and delay the temporal appearance of radiation-induced MN-PCE in the bone marrow [53,54,56]. The loss of linearity at high doses is ascribed to cell cycle effects and under-representation of damaged cells in the PCE pool present at the time of harvesting, potentially due to apoptosis of heavily damaged erythroid precursors [53,54,56,57,61,64].

Our measurement of 0.46% for MN-PCE in bone marrow of unirradiated adult female C57BL/6 mice was somewhat lower than previously reported results for spontaneous MN levels in marrow of inbred laboratory mice, which are typically reported as between 2% and 3% [51,65,66]. It is known that assay method (i.e., automated detection by flow cytometry versus manual microscopic scoring), sex, variation between individual mice and age can all affect reported outcomes. Our γ radiation dose–response data indicates that a 20 mGy exposure is not sufficiently damaging to increase MN-PCE frequencies in C57BL/6 mice above baseline levels.

A linear dose–response relationship for the induction of C57BL/6 mouse bone marrow MN-PCE was supported by our experimental data for doses between 100 mGy and 1 Gy. Departure from linearity was observed at doses between 1 and 2 Gy, with saturation observed at doses of ≥1 Gy. This departure from linearity at high doses may be due to a delay in the sampling time required for the detection of the maximum MN-PCE frequency at these doses. Acute high doses of radiation ≥500 mGy had a cytotoxic effect on bone marrow erythroblast proliferation/maturation of C57BL/6 mice, without an observed threshold of maximal effect for doses up to 2 Gy. The 100 mGy radiation exposure, while increasing MN-PCE frequency in C57BL/6 mice, did not have a cytotoxic effect on bone marrow erythrocyte proliferation, consistent with previous observations [57].

Our temporal analysis of radiation-induced DNA damage following a single 2 Gy acute radiation exposure revealed that bone marrow MN-PCE frequencies were highest in C57BL/6 mice 48 h post-irradiation and began to decline steeply between 54 and 72 h post-irradiation. Since kinetic analysis was not performed for lower dose exposures, it is not clear whether the maximum peak at 48 h post-irradiation is delayed by perturbation of the cell cycle, but this is a likely scenario since *P*/*N* ratios in 2 Gy-irradiated mice were depressed as early as 18 h post-irradiation and did not start to rise again until 72 h post-irradiation.

C57BL/6 mice are considered radio-resistant relative to other strains [67,68]. Radio-sensitive BALB/c mice are less efficient in DSB rejoining due to a polymorphism in the gene encoding DNA-dependent protein kinase catalytic subunit (DNA-PKcs) [69,70]. BALB/c mice are susceptible to developing solid tumours following acute radiation exposures and exhibit increased radiation-induced DNA damage and genomic instability relative to C57BL/6 mice and other strains [67,68,71,72,73,74,75,76]. Splenocyte MN frequencies were found to be significantly higher in BALB/c mice than for C57BL/6 mice following in vivo or in vitro 2 Gy γ irradiation in our previous analysis [43]. In the current study, MN-PCE frequencies in BALB/c mice were similar to those of C57BL/6 mice following 2 Gy radiation exposure, revealing that BALB/c mouse bone marrow cells were not radio-sensitive in comparison to C57BL/6 mice at this dose. Bone marrow *P*/*N* ratios were also similar in the two strains (i.e., a 50% to 60% decrease from control ratios) following 2 Gy irradiation. Unlike the situation for C57BL/6 mice, where the 20 mGy radiation exposure had no effect on MN-PCE frequencies, a modest but significant increase (1.7-fold) above the spontaneous MN-PCE frequency was observed following 20 mGy irradiation of BALB/c mice, indicating a potentially lower dose threshold for the induction of cytogenetic damage in BALB/c bone marrow in comparison to C57BL/6 mice. A complete dose–response and kinetic induction profile for MN-PCE for intermediate doses of radiation in BALB/c mice is required to fully address strain differences for the bone marrow MN-PCE assay. The kinetic profiles of MN-PCE induction and bone marrow *P*/*N* ratio depression in irradiated BALB/c mice were similar to C57BL/6 mice. For both strains of mice, turnover of MN-PCEwas much more rapid than that previously observed for spleen cells [43], reflecting the high proliferative index of bone marrow progenitor cells.

Mitigation of high-dose acute radiation-induced DNA damage (manifested as reduced frequencies of micronucleated erythrocytes or chromosome/chromatid cytogenetic aberrations) has been documented in bone marrow cells of mice exposed to chronic [77] or acute low-dose γ or X-ray radiation [18,27,78]. Cytogenetic radio-adaptive responses have also been described in bone marrow cells following cross-adaptation experiments in which mice were exposed to non-ionizing radiofrequency (RF) fields [79,80] or other radio-protective compounds such as melanin [81] prior to exposure to acute challenging doses of radiation.

We examined potential radio-adaptive responses for reduction of radiation-induced cytogenetic damage or protection from radiation-induced suppression of *P*/*N* ratio in bone marrow of C57BL/6 and BALB/c mice. Radio-adaptive responses for these endpoints could potentially arise due to a decrease in the number of cellular DNA double strand breaks, increased DNA repair proficiency, an increase in cellular proliferative index, or increased apoptosis of erythroblasts or PCEs harbouring DNA damage. Based on the duration of the erythrocyte cell cycle [51], the γ priming doses in our study may have targeted the same erythroblast generation (likely for the experiments with a four hour interval between priming and challenge doses) or preceding erythroblast generations (likely for the 24 h time periods and multiple 20 mGy priming regimes) of those cells exposed to the 2 Gy acute radiation dose.

Our results clearly indicate the absence of radio-adaptive responses for radiation-induced cytogenetic damage or radiation-induced suppression of erythrocyte proliferation/maturation index in bone marrow erythrocytes of γ-irradiated C57BL/6 and BALB/c mice, as we failed to observe statistically significant changes in either MN-PCE frequencies or *P*/*N* ratios in mice that received low-dose priming radiation exposures. In the first 30 h post-radiation a modest potential additive effect (a 1.1- to 1.2-fold increase in MN-PCE frequencies) was observed for C57BL/6 mice that received a 20 mGy priming dose in comparison to mice receiving only the acute 2 Gy dose, with statistical significance between the two treatment groups for the 18-h time point. A trend for lower *P*/*N* ratios (although not statistically significant) was also observed for mice that received a 20 mGy priming dose in relation to those that received only 2 Gy radiation for the time period 24 to 48 h post-radiation. The trend for decreased *P*/*N* ratio and increased MN-PCE frequencies in C57BL/6 mice receiving a priming dose indicates that the priming dose may have had a small effect on impeding the turnover of immature erythrocytes in the bone marrow of mice and the clearance of PCEs with DNA damage in the first 48 h following a high dose acute radiation exposure. Radio-adaptive kinetic analysis was not performed for the BALB/c strain in our study.

This study is an extension of our previous study of the cytotoxic effects of low-dose radiation on mouse spleen cells [43]. Considered together, the results of both studies represent a multiple tissue, multiple endpoint approach to exploring links between the induction and repair of cytogenetic damage in haematopoietic cells, genomic instability and progression to haematological cancer [82,83]. Our findings support radio-sensitivity of BALB/c mice for DNA damage in spleen lymphocytes and also potentially indicate a lower threshold for the induction of cytogenetic damage in BALB/c bone marrow erythrocytes. We failed to observe a radio-adaptive response for radiation-induced cytogenetic damage or suppression of erythrocyte maturation/proliferation in bone marrow of either mouse strain, supporting our previous indications that that low-dose radiation exposures leading to reduced carcinogenic potential may not be related to alterations in short-term cytogenetic damage [43]. The lack of radio-adaptive response for cytogenetic and DSB-related endpoints observed in lymphoid/haematological tissues of mice following low-dose radiation in this and our previous studies indicates a requirement to investigate pathways other than DNA DSB repair as underlying radio-adaptive responses in relation to reduced latency for haematological cancers. Results from our laboratory and others clearly implicate *p53* as an important oncogene in the modulation of tumourigenesis in mice following low-dose radiation. *p53*-regulated pathways and processes other than the canonical DNA damage response are now recognized as being important for tumourigenesis, including metabolism, stem cell maintenance and tumour microenvironment [84]. We are currently investigating repair of DNA lesions other than DSBs, such as mismatch repair (MMR), base excision repair (BER) and nucleotide excision repair (NER) in radio-adaptive responses, as well as the role of low-dose radiation in regulating inflammation and immunity, epigenetics and stem cell homeostasis in laboratory mice exposed to low-dose ionizing radiation.

## 4. Materials and Methods

### 4.1. Mice

Female C57BL/6J and BALB/cJ mice were purchased from the Jackson Laboratory (Bar Harbor, ME, USA). Mice were housed in the specific pathogen-free Biological Research Facility (BRF) at Canadian Nuclear Laboratories (CNL; Chalk River, ON, Canada). Mice were housed in either single or duplex cages (Thoren Caging Systems Inc., Hazelton, PA, USA) and fed ad libitum with Charles River Rodent Chow (Frederick, MD, USA). Experimental protocols (as part of Animal Protocol BRF 06-03, approved 17 August 2006) received prior approval from CNL’s Animal Care Committee and were performed in accordance with the guidelines of the Canadian Council on Animal Care. Experiments were conducted using adult female mice between two to five months of age. Mice were allowed to acclimatize in the BRF for at least two weeks prior to irradiation or sham irradiation. Mice were maintained in an environment with constant temperature (23 °C), air ventilation and 12 h light/dark cycle. Mice were routinely tested for pathogens to confirm their pathogen-free status.

### 4.2. Irradiations

An open beam ^60^Co-γ source (γBeam 150, Hopewell Designs Inc., Alpharetta, GA, USA) was used to deliver whole body low-dose, low-dose-rate priming doses (20 mGy and 100 mGy at dose rates of 0.5 mGy/min and 10 mGy/min, respectively) and intermediate doses (500 mGy at a dose rate of 10 mGy/min) to mice. For exposures, unrestrained mice in plastic cages were exposed to the beam source. For whole body acute high-dose in vivo 1 and 2 Gy irradiations, unrestrained mice individually housed in irradiation vessels were exposed in an enclosed ^60^Co-γ source irradiator (γCell 220, MDS Nordion^TM^ Inc., Ottawa, ON, Canada) at dose rates of ~150 mGy/min to 174 mGy/min.

### 4.3. Bone Marrow MN-PCE Frequency and P/N Ratio Determination

Mice were euthanized by cervical dislocation. Cytogenetic analysis of MN in bone marrow PCEs was conducted using standardized procedures [45,85,86]. The femoral bones of each mouse were excised and the bone marrow was aspirated by flushing of the femoral bones with 100 μL of foetal bovine serum (FBS; Sigma-Aldrich^®^, St. Louis, MO, USA) using a 27-gauge needle and a 1 mL syringe. Marrow cells from each mouse were flushed into a single well of a 96-well microplate. The bone marrow cells were gently mixed and a drop of the resulting cell suspension was then smeared onto a pre-cleaned glass slide to create a monolayer of bone marrow cells. Cell preparations were dried overnight, fixed in 100% methanol (Fisher Scientific, Pittsburgh, PA, USA) and stored at room temperature prior to staining and analysis. Cells were stained for 15 s in Acridine Orange (25 μg/mL; Fisher Scientific, Pittsburgh, PA, USA) dissolved in Gurr Buffer, pH 6.8. Slides were washed three times in Gurr Buffer. Two slides were prepared from each animal. Stained cells were analyzed by fluorescence microscopy (using a 63× oil objective lens) for determination of MN-PCE frequency and differentiation of immature PCEs and mature NCEs. One thousand PCEs were scored on each slide (two thousand total PCE per animal) to calculate MN frequency. MN-PCE frequencies were calculated as (total number of MN scored/1000 PCEs) × 100 and reported as MN-PCE% (mean pooled value for all mice in treatment cohort ± standard error). Three hundred erythrocytes per slide (six hundred total cells per animal) were scored for determination of the *P*/*N* ratio, calculated as (Number of PCEs)/(Total number of PCEs + NCEs) and reported as *P*/*N* (mean pooled value for all mice in treatment cohort ± standard error).

### 4.4. Statistical Analysis

For each experimental or control animal group, statistical outliers identified using Grubb’s test (significance level 0.05) were excluded from statistical analysis. Statistical comparison of treatment groups was carried out using the unpaired, two-tailed Student’s *t*-test. Dose–response curve fit and regression analysis was performed using Microsoft Excel 2013.

## References

[1] 1990 Recommendations of the International Commission on Radiological Protection. http://www.ncbi.nlm.nih.gov/pubmed/2053748.

[2] ICRP Statement on Tissue Reactions and Early and Late Effects of Radiation in Normal Tissues and Organs: Threshold Doses for Tissue Reactions in a Radiation Protection Context. http://www.sciencedirect.com/science/article/pii/S0146645312000024.

[3] Low-Dose Extrapolation of Radiation-Related Cancer Risk. http://www.icrp.org/publication.asp?id=ICRP%20Publication%2099.

[4] Mothersill C., Seymour C. (2014). Implications for human and environmental health of low-doses of ionising radiation. J. Environ. Radioact..

[5] Kadhim M., Salomaa S., Wright E., Hildebrandt G., Belyakov O.V., Prise K.M., Little M.P. (2012). Non-targeted effects of ionising radiation-Implications for low-dose risk. Mutat. Res. Rev. Mutat..

[6] Averbeck D. (2010). Non-targeted effects as a paradigm breaking evidence. Mutat. Res. Fundam. Mol. Mech. Mutagenes..

[7] Matsumoto H., Tomita M., Otsuka K., Hatashita M. (2009). A new paradigm in radioadaptive response developing from microbeam research. J. Radiat. Res..

[8] Calabrese E.J., O’Connor M.K. (2014). Estimating risk of low radiation doses—A critical review of the BEIR VII report and its use of the linear no-threshold (LNT) hypothesis. Radiat. Res..

[9] Seong K.M., Seo S., Lee D., Kim M.J., Lee S.S., Park S., Jin Y.W. (2016). Is the Linear No-threshold dose-response paradigm still necessary for the assessment of health effects of low-dose radiation?. J. Korean Med. Sci..

[10] Dauer L.T., Brooks A.L., Hoel D.G., Morgan W.F., Stram D., Tran P. (2010). Review and evaluation of updated research on the health effects associated with low-dose ionising radiation. Radiat. Prot. Dosim..

[11] Sacks B., Meyerson G., Siegel J.A. (2016). Epidemiology without biology: False paradigms, unfounded assumptions, and specious statistics in radiation science (with commentaries by Inge Schmitz-Feuerhake and Christopher Busby and a reply by the authors). Biol. Theory.

[12] Wolff S. (1998). The adaptive response in radiobiology: Evolving insights and implications. Environ. Health Perspect..

[13] Matsumoto H., Hamada N., Takahashi A., Kobayashi Y., Ohnishi T. (2007). Vanguards of paradigm shift in radiation biology: Radiation-induced adaptive and bystander responses. J. Radiat. Res..

[14] Tapio S., Jacob V. (2007). Radioadaptive response revisited. Radiat. Environ. Biophys..

[15] Zhao Y., Zhong R., Sun L., Jia J., Ma S., Liu X. (2015). Ionizing radiation-induced adaptive response in fibroblasts under both monolayer and 3-dimensional conditions. PLoS ONE.

[16] Park H.S., You G.E., Yang K.H., Kim J.Y., An S., Song J.Y., Lee S.J., Lim Y.K., Nam S.Y. (2015). Role of AKT and ERK pathways in controlling sensitivity to ionizing radiation and adaptive response induced by low-dose radiation in human immune cells. Eur. J. Cell Biol..

[17] Toprani S.M., Das B. (2015). Radio-adaptive response of base excision repair genes and proteins in human peripheral blood mononuclear cells exposed to γ radiation. Mutagenesis.

[18] Cai L., Liu S.Z. (1990). Induction of cytogenetic adaptive response of somatic and germ cells in vivo and in vitro by low-dose X-irradiation. Int. J. Radiat. Biol..

[19] Wolff S., Afzal V., Wiencke J.K., Olivieri G., Michaeli A. (1988). Human lymphocytes exposed to low-doses of ionizing radiations become refractory to high doses of radiation as well as to chemical mutagens that induce double-strand breaks in DNA. Int. J. Radiat. Biol..

[20] Shadley J.D., Afzal V., Wolff S. (1987). Characterization of the adaptive response to ionizing radiation induced by low-doses of X rays to human lymphocytes. Radiat. Res..

[21] Azzam E.I., Raaphorst G.P., Mitchel R.E. (1994). Radiation-induced adaptive response for protection against micronucleus formation and neoplastic transformation in C3H 10T1/2 mouse embryo cells. Radiat. Res..

[22] Zaichkina S.I., Dyukina A.R., Rozanova O.M., Simonova N.B., Romanchenko S.P., Sorokina S.S., Zakrzhevskaya D.T., Yusupov V.I., Bagratashvili V.N. (2016). Induction of the adaptive response in mice exposed to He-Ne laser and X-ray radiation. Bull. Exp. Biol. Med..

[23] Premkumar K., Shankar B.S. (2016). Involvement of MAPK signalling in radioadaptive response in BALB/c mice exposed to low-dose ionizing radiation. Int. J. Radiat. Biol..

[24] Lacoste-Collin L., Jozan S., Pereda V., Courtade-Saidi M. (2015). Influence of a continuous very low-dose of γ-rays on cell proliferation, apoptosis and oxidative stress. Dose Response.

[25] Grdina D.J., Murley J.S., Miller R.C., Mauceri H.J., Sutton H.G., Thirman M.J., Li J.J., Woloschak G.E., Weichselbaum R.R. (2013). A manganese superoxide dismutase (SOD2)-mediated adaptive response. Radiat. Res..

[26] Day T.K., Zeng G., Hooker A.M., Bhat M., Turner D.R., Sykes P.J. (2007). Extremely low-doses of X-radiation can induce adaptive responses in mouse prostate. Dose Response.

[27] Farooqi Z., Kesavan P.C. (1993). Low-dose radiation-induced adaptive response in bone marrow cells of mice. Mutat. Res. Lett..

[28] Phan N., de Lisio M., Parise G., Boreham D.R. (2012). Biological effects and adaptive response from single and repeated computed tomography scans in reticulocytes and bone marrow of C57BL/6 mice. Radiat. Res..

[29] Mitchel R.E., Jackson J.S., McCann R.A., Boreham D.R. (1999). The adaptive response modifies latency for radiation-induced myeloid leukemia in CBA/H mice. Radiat. Res..

[30] Mitchel R.E., Jackson J.S., Morrison D.P., Carlisle S.M. (2003). Low-doses of radiation increase the latency of spontaneous lymphomas and spinal osteosarcomas in cancer-prone, radiation-sensitive *Trp53* heterozygous mice. Radiat. Res..

[31] Ishii K., Hosoi Y., Yamada S., Ono T., Sakamoto K. (1996). Decreased incidence of thymic lymphoma in AKR mice as a result of chronic, fractionated low-dose total-body X irradiation. Radiat. Res..

[32] Hosoi Y., Sakamoto K. (1993). Suppressive effect of low-dose total body irradiation on lung metastasis: Dose dependency and effective period. Radiother. Oncol..

[33] Ina Y., Tanooka H., Yamada T., Sakai K. (2005). Suppression of thymic lymphoma induction by life-long low-dose-rate irradiation accompanied by immune activation in C57BL/6 mice. Radiat. Res..

[34] Ina Y., Sakai K. (2004). Prolongation of life span associated with immunological modification by chronic low-dose-rate irradiation in MRL-lpr/lpr mice. Radiat. Res..

[35] Yonezawa M., Takeda A., Misonoh J. (1990). Acquired radioresistance after low-dose X-irradiation in mice. J. Radiat. Res..

[36] Mitchel R.E., Burchart P., Wyatt H. (2008). A lower dose threshold for the in vivo protective adaptive response to radiation. Tumorigenesis in chronically exposed normal and *Trp53* heterozygous C57BL/6 mice. Radiat. Res..

[37] Mitchel R.E., Jackson J.S., Carlisle S.M. (2004). Upper dose thresholds for radiation-induced adaptive response against cancer in high-dose-exposed, cancer-prone, radiation-sensitive *Trp53* heterozygous mice. Radiat. Res..

[38] Hooker A.M., Bhat M., Day T.K., Lane J.M., Swinburne S.J., Morley A.A., Sykes P.J. (2004). The linear no-threshold model does not hold for low-dose ionizing radiation. Radiat. Res..

[39] James S.J., Enger S.M., Makinodan T. (1991). DNA strand breaks and DNA repair response in lymphocytes after chronic in vivo exposure to very low-doses of ionizing radiation in mice. Mutat. Res. Fundam. Mol. Mech. Mutagenes..

[40] Liu S.Z., Cai L., Sun J.B. (1990). Effect of low-dose radiation on repair of DNA and chromosome damage. Acta Biol. Hung..

[41] Schwartz J.L. (2007). Variability: The common factor linking low-dose-induced genomic instability, adaptation and bystander effects. Mutat. Res. Fundam. Mol. Mech. Mutagenes..

[42] Blimkie M.S., Fung L.C., Petoukhov E.S., Girard C., Klokov D. (2014). Repair of DNA double-strand breaks is not modulated by low-dose γ radiation in C57BL/6J mice. Radiat. Res..

[43] Bannister L.A., Serran M.L., Mantha R.R. (2015). Low-dose γ radiation does not induce an adaptive response for micronucleus induction in mouse splenocytes. Radiat. Res..

[44] Heddle J.A. (1973). A rapid in vivo test for chromosomal damage. Mutat. Res. Fundam. Mol. Mech. Mutagenes..

[45] Schmid W. (1975). The micronucleus test. Mutat. Res./Environ. Mutagenes. Relat. Subj..

[46] Test No. 475: Mammalian Bone Marrow Chromosomal Aberration Test. http://www.oecd-ilibrary.org/environment/test-no-475-mammalian-bone-marrow-chromosome-aberrationtest_9789264071308-en.

[47] Heddle J.A., Hite M., Kirkhart B., Mavournin K., MacGregor J.T., Newell G.W., Salamone M.F. (1983). The induction of micronuclei as a measure of genotoxicity. A report of the US environmental protection agency gene-tox program. Mutat. Res. Rev. Genet. Toxicol..

[48] Feinendegen L.E., Pollycove M., Sondhaus C.A. (2004). Responses to low-doses of ionizing radiation in biological systems. Nonlinearity Biol. Toxicol. Med..

[49] Feinendegen L.E., Pollycove M., Neumann R.D. (2007). Whole-body responses to low-level radiation exposure: New concepts in mammalian radiobiology. Exp. Hematol..

[50] Adler I.D. (1984). Cytogenetic Tests in Mammals.

[51] Mavournin K.H., Blakey D.H., Cimino M.C., Salamone M.F., Heddle J.A. (1990). The in vivo micronucleus assay in mammalian bone marrow and peripheral blood. A report of the US environmental protection agency gene-tox program. Mutat. Res. Rev. Genet. Toxicol..

[52] Krishna G., Hayashi M. (2000). In vivo rodent micronucleus assay: Protocol, conduct and data interpretation. Mutat. Res. Fundam. Mol. Mech. Mutagenes..

[53] Jenssen D., Ramel C. (1978). Factors affecting the induction of micronuclei at low-doses of X-rays, MMS and dimethylnitrosamine in mouse erythroblasts. Mutat. Res. Genet. Toxicol..

[54] Cole R.J., Taylor N., Cole J., Arlett C.F. (1981). Short-term tests for transplacentally active carcinogens. I. Micronucleus formation in fetal and maternal mouse erythroblasts. Mutat. Res./Fundam. Mol. Mech. Mutagenes..

[55] Hart J.W., Hartley-Asp B. (1983). Induction of micronuclei in the mouse: Revised timing of the final stage of erythropoiesis. Mutat. Res. Lett..

[56] Devi P.U., Sharma A.S. (1990). Mouse bone-marrow response to low-doses of whole-body γ irradiation: Induction of micronuclei. Int. J. Radiat. Biol..

[57] Abramsson-Zetterberg L., Zetterberg G., Grawe J. (1996). The time-course of micronucleated polychromatic erythrocytes in mouse bone marrow and peripheral blood. Mutat. Res./Fundam. Mol. Mech. Mutagenes..

[58] Cornforth M.N., Goodwin E.H. (1991). Transmission of radiation-induced acentric chromosomal fragments to micronuclei in normal human fibroblasts. Radiat. Res..

[59] Osipov A.N., Klokov D.Y., Elakov A.L., Rozanova O.M., Zaichkina S.I., Aptikaeva G.F., Akhmadieva A.K. (2004). Comparison in vivo study of genotoxic action of high-versus very low dose-rate γ-irradiation. Nonlinearity Biol. Toxicol. Med..

[60] Kumar M.S., Unnikrishnan M.K., Devi P.U. (2003). Effect of 5-aminosalicylic acid on radiation-induced micronuclei in mouse bone marrow. Mutat. Res. Fundam. Mol. Mech. Mutagenes..

[61] Jagetia G.C., Ganapathi N.G. (1994). Radiation-induced micronucleus formation in mouse bone marrow after low-dose exposures. Mutat. Res./Fundam. Mol. Mech. Mutagenes..

[62] Mozdarani H., Gharbali A. (1993). Radioprotective effects of cimetidine in mouse bone marrow cells exposed to γ-rays as assayed by the micronucleus test. Int. J. Radiat. Biol..

[63] Zetterberg G., Grawe J. (1993). Flow cytometric analysis of micronucleus induction in mouse erythrocytes by γ-irradiation at very low-dose-rates. Int. J. Radiat. Biol..

[64] Dertinger S.D., Tsai Y., Nowak I., Hyrien O., Sun H., Bemis J.C., Torous D.K., Keng P., Palis J., Chen Y. (2007). Reticulocyte and micronucleated reticulocyte responses to γ irradiation: Dose-response and time-course profiles measured by flow cytometry. Mutat. Res. Genet. Toxicol. Environ. Mutagenes..

[65] Salamone M.F., Mavournin K.H. (1994). Bone marrow micronucleus assay: A review of the mouse stocks used and their published mean spontaneous micronucleus frequencies. Environ. Mol. Mutagenes..

[66] (1986). Sex difference in the micronucleus test. The collaborative study group for the micronucleus Test. Mutat. Res..

[67] Grahn D., Hamilton K.F. (1957). Genetic variation in the acute lethal response of four inbred mouse strains to whole body X-irradiation. Genetics.

[68] Yuhas J.M., Storer J.B. (1969). On mouse strain differences in radiation resistance: Hematopoietic death and the endogenous colony-forming unit. Radiat. Res..

[69] Okayasu R., Suetomi K., Yu Y., Silver A., Bedford J.S., Cox R., Ullrich R.L. (2000). A deficiency in DNA repair and DNA-PKcs expression in the radiosensitive BALB/c mouse. Cancer Res..

[70] Yu Y., Okayasu R., Weil M.M., Silver A., McCarthy M., Zabriskie R., Long S., Cox R., Ullrich R.L. (2001). Elevated breast cancer risk in irradiated BALB/c mice associates with unique functional polymorphism of the Prkdc (DNA-dependent protein kinase catalytic subunit) gene. Cancer Res..

[71] Roderick T.H. (1963). The response of twenty-seven inbred strains of mice to daily doses of whole-body X-irradiation. Radiat. Res..

[72] Storer J.B., Mitchell T.J., Fry R.J. (1988). Extrapolation of the relative risk of radiogenic neoplasms across mouse strains and to man. Radiat. Res..

[73] Rithidech K.N., Udomtanakunchai C., Honikel L.M., Whorton E.B. (2012). No evidence for the in vivo induction of genomic instability by low doses of 137Cs γ rays in bone marrow cells of BALB/CJ and C57BL/6J mice. Dose Response.

[74] Ullrich R.L., Davis C.M. (1999). Radiation-induced cytogenetic instability in vivo. Radiat. Res..

[75] Ponnaiya B., Cornforth M.N., Ullrich R.L. (1997). Radiation-induced chromosomal instability in BALB/c and C57BL/6 mice: The difference is as clear as black and white. Radiat. Res..

[76] Hamasaki K., Imai K., Hayashi T., Nakachi K., Kusunoki Y. (2007). Radiation sensitivity and genomic instability in the hematopoietic system: Frequencies of micronucleated reticulocytes in whole-body X-irradiated BALB/c and C57BL/6 mice. Cancer Sci..

[77] Fomenko L.A., Kozhanovskaia K., Gaziev A.I. (1991). Micronucleus formation in the bone marrow cells of chronically irradiated mice with subsequent acute γ irradiation. Radiobiologiia.

[78] Zhang L. (1995). Cytogenetic adaptive response induced by pre-exposure in human lymphocytes and marrow cells of mice. Mutat. Res. Genet. Toxicol. Environ. Mutagenes..

[79] Cao Y., Xu Q., Jin Z.D., Zhou Z., Nie J.H., Tong J. (2011). Induction of adaptive response: Pre-exposure of mice to 900 MHz radiofrequency fields reduces hematopoietic damage caused by subsequent exposure to ionising radiation. Int. J. Radiat. Biol..

[80] Jiang B., Zong C., Zhao H., Ji Y., Tong J., Cao Y. (2013). Induction of adaptive response in mice exposed to 900MHz radiofrequency fields: Application of micronucleus assay. Mutat. Res. Genet. Toxicol. Environ. Mutagenes..

[81] Mosse I., Kostrova L., Subbot S., Maksimenya I., Molophei V. (2000). Melanin decreases clastogenic effects of ionizing radiation in human and mouse somatic cells and modifies the radioadaptive response. Radiat. Environ. Biophys..

[82] Watson G.E., Pocock D.A., Papworth D., Lorimore S.A., Wright E.G. (2001). In vivo chromosomal instability and transmissible aberrations in the progeny of haemopoietic stem cells induced by high- and low-LET radiations. Int. J. Radiat. Biol..

[83] Mukherjee D., Coates P.J., Lorimore S.A., Wright E.G. (2012). The in vivo expression of radiation-induced chromosomal instability has an inflammatory mechanism. Radiat. Res..

[84] Bieging K.T., Mello S.S., Attardi L.D. (2014). Unravelling mechanisms of *p53*-mediated tumour suppression. Nat. Rev. Cancer.

[85] Tinwell H., Ashby J. (1989). Comparison of acridine orange and Giemsa stains in several mouse bone marrow micronucleus assays—Including a triple dose study. Mutagenesis.

[86] Hayashi M., Sofuni T., Ishidate M. (1983). An application of Acridine Orange fluorescent staining to the micronucleus test. Mutat. Res. Lett..

